# Factors Associated with Continued Food Insecurity among Households Recovering from Hurricane Katrina

**DOI:** 10.3390/ijerph15081647

**Published:** 2018-08-03

**Authors:** Lauren A. Clay, Mia A. Papas, Kimberly B. Gill, David M. Abramson

**Affiliations:** 1 Health Services Administration, D’Youville College, Buffalo, NY 14201, USA; 2 Christiana Care Health System, Value Institute, Wilmington, DE 19899, USA; mia.papas@christianacare.org; 3 Disaster Research Center, University of Delaware, Newark, DE 19716, USA; kgill@udel.edu; 4 College of Global Public Health, New York University, New York, NY 10012, USA; david.abramson@nyu.edu

**Keywords:** food insecurity, disaster, family health, Hurricane Katrina, mental health, physical health, social support

## Abstract

In 2010, 14.5% of US households experienced food insecurity, which adversely impacts health. Some groups are at increased risk for food insecurity, such as female-headed households, and those same groups are often also at increased risk for disaster exposure and the negative consequences that come with exposure. Little research has been done on food insecurity post-disaster. The present study investigates long-term food insecurity among households heavily impacted by Hurricane Katrina. A sample of 683 households participating in the Gulf Coast Child and Family Health Study were examined using a generalized estimation model to determine protective and risk factors for food insecurity during long-term recovery. Higher income (Odds Ratio (OR) 0.84, 95% Confidence Interval (CI) 0.77, 0.91), having a partner (OR 0.93; 95% CI 0.89, 0.97), or “other” race were found to be protective against food insecurity over a five-year period following disaster exposure. Low social support (OR 1.14; 95% CI 1.08, 1.20), poor physical health (OR 1.08; 95% CI 1.03, 1.13) or mental health (OR 1.13; 95% CI 1.09, 1.18), and female sex (OR 1.05; 95% CI 1.01, 1.10) were risk factors. Policies and programs that increase access to food supplies among high-risk groups are needed to reduce the negative health impacts of disasters.

## 1. Introduction

In 2010, 14.5 percent of households in the United States experienced food insecurity, an increase from 11.0 percent in 2005 and 10.9 percent in 2006. Furthermore, 9.8 percent of households with children experienced food insecurity at some point during 2010, affecting 3.9 million households [1].

Food insecurity is higher than the national average for households with children, headed by a single adult, with low income, in rural or urban areas, for minorities, and those residing in the South region of the US [1,2]. The United States Department of Agriculture (USDA) reports that food insecurity is three times more prevalent in single-female-headed households compared to households headed by married couples [3,4]. Furthermore, food insecurity is more than twice as likely in households headed by Hispanic or Black individuals than those households headed by non-Hispanic whites [2]. Food insecurity in the South in 2010 was 10.4 percent, higher than the West (9.4 percent), Midwest (8.1 percent) and Northeast (7.7 percent) regions [1]. In addition to socio-economic factors, a caregiver with poor physical and mental health, disability, weaker social ties and emotional support, and changes in housing or income stability are risk factors for child food insecurity [5,6,7,8,9].

Many research studies have demonstrated that children in food insecure households are at risk for adverse physical and mental health consequences, such as behavioral problems, lower educational achievement, psychosocial dysfunction, depressive symptoms, suicidal symptoms, anxiety, and chronic health conditions [10,11,12,13,14,15,16]. A recent literature review completed by Gunderson and colleagues found that food-insecure children are more likely to experience “anemia, lower nutrient intake, cognitive problems, higher levels of aggression and anxiety, poorer general health, poorer oral health, and higher risk of being hospitalized, having asthma, having some birth defects, or experiencing behavioral problems” [17]. 

Even though there are a number of assistance programs to increase nutritious food intake among those at risk, such as the Special Supplemental Feeding Program for Women, Infant, and Children (WIC), the Supplemental Nutrition Assistance Program (SNAP), and the National School Lunch Program (NSLP), food insecurity remains high in the United States, due at least in part to the lack of understanding about the causes of food insecurity and lack of evidence for effective policy and program solutions [17]. Gaps in research on food insecurity remain. While research shows that disability influences food security, for example, little research has investigated how disability is associated with food insecurity risk. Many studies of risk and protective factors use nationally representative samples in the United States; little research has focused on overlooked groups or special populations outside of traditional demographic groups. Policy solutions are likely to look different for specific populations, such as those that have experienced a significant disaster event. Long-term data collection has also been called for to better understand how food insecurity changes over time, as well as studies that incorporate qualitative methods and the voices of children to more fully tell the story of food insecurity in the U.S. [17,18,19].

Few studies have focused on food insecurity post-disaster in the United States. Programs have been implemented to aid food-insecure populations after disaster, such as modifications to allowable purchases for Supplemental Nutrition Assistance Program (SNAP) beneficiaries after Superstorm Sandy in New Jersey so families were able to repurchase lost food supplies [20], however little is understood about those that are living on the cusp of food insecurity that may be pushed into insecurity due to the disruption of a disaster or other change in circumstance, such as changes in housing or decline in mothers’ mental health [9].

We know that vulnerability to disaster exposure and negative consequences vary based on resource access, age, physical ability, sex, race and ethnicity, and living conditions [21,22,23]. We also know that single women, single mothers, and caregivers, in addition to experiencing increased risk of food insecurity, are also more vulnerable following disasters [3,4,24], and race, ethnicity, disability, functional and access needs, and mental health contribute to decreased disaster preparedness and may impede or slow disaster recovery [25,26,27,28,29,30,31]. Following disaster exposure, resources are lost, including material (personal property), social (social support), and neighborhood-based resources due to relocation, and food insecurity is common or the odds of food insecurity increase [32,33,34,35,36]. Resource loss contributes to psychological distress, such as anxiety, depression, and post-traumatic stress disorder (PTSD) [33,34,37]. This loops back to the influence of caretaker mental health on child food security.

Factors contributing to food insecurity and disaster risk are complex, and the impact from each influences health outcomes. However, few studies have explored food insecurity in a post-disaster setting. In summary, prior research has established the impact of food insecurity on health and well-being, the noted risk factor of changes in housing and economic circumstance on food insecurity risk, the need for longitudinal study of food insecurity, and the evidence of increased food insecurity risk post-Hurricane Katrina. Given this, the present study examines long-term food insecurity in a sample of households heavily impacted by Hurricane Katrina, taking into account resource loss and demographic characteristics.

## 2. Materials and Methods

### 2.1. Sample and Data Collection

Households were surveyed as part of the Gulf Coast Child and Family Health (G-CAFH) Study, a longitudinal study of household disaster recovery following Hurricane Katrina in Louisiana and Mississippi. Households were recruited in 2006 and participated in an annual follow up. Households were randomly sampled from census blocks classified by Federal Emergency Management Agency (FEMA) assessments as moderately to extensively damaged, and from FEMA subsidized housing. The current analysis examined data from households that participated in waves two, three, and four of the G-CAFH Study. Wave two was collected between May and July 2007 (*n* = 803), wave three was collected between June and August 2008 (*n* = 777), and wave four was collected between October 2009 and March 2010 (*n* = 844), resulting in a four-year observation period for 683 households. A bias analysis conducted by the G-CAFH Study team demonstrated that there are no significant differences due to attrition between the cohort at wave one and at wave four. Additional information on study design and methodology has been published elsewhere [38,39].

### 2.2. Measures

Food insecurity was assessed in waves two, three, and four of the study by asking participants to think about their basic needs over the past three to six months. In wave two, respondents were asked to report on the past six months, “How well has your need for food for the household been met?” (not met, somewhat met, or met completely), and in waves three and four, respondents were asked, “In the past three months, how often it has happened there was not enough money in the household for food that you (the family) should have?” (never, once in a while, fairly often, or very often). Respondents were classified as food insecure in each wave if they answered that the need was not met or they fairly or very often did not have enough money in the household for food. The United States Department of Agriculture defines food insecurity as “a household-level economic and social condition of limited or uncertain access to adequate food [40].” The G-CAFH study questions are intended to capture social and economic limitations to access adequate food. Although not validated against the USDA measure for food insecurity, these questions provide a starting point to understand food insecurity in a disaster-affected population.

Social support was assessed by asking respondents if they had someone they could count on for everyday favors, such as borrowing a little money, to care for you if you were confined to a bed for several weeks, to lend you money for a medical emergency, to talk to about family relationship troubles, or to help you find housing if you had to move. Respondents were categorized as having low social support if they responded yes to fewer than two of these statements.

Physical and mental health were self-reported by respondents using the Short Form (SF)-12 Health Survey [41]. The Mental Component Score (MCS) and Physical Component Score (PCS) were computed. A PCS score of less than 45 was classified as Physical Health Distress, and an MCS score of less than 42 was classified as Mental Health Distress, consistent with past research [41,42]. Respondents were classified as having a disability if they responded “disabled” when reporting on characteristics of the household.

Demographic variables included in this analysis included income (<$10 K, $10–20 K, $20–35 K, $35–50 K, >$50 K), age (18–34, 35–49, 50–65, 66+), race and ethnicity (Black, White, Latino, other), and sex (male, female) and were self-reported by G-CAFH participants.

### 2.3. Statistical Analysis

Generalized estimating equations were used to determine bivariate associations between each exposure variable and our outcome over time. In addition, this longitudinal modeling strategy was employed to examine multivariate associations between exposures and food insecurity after adjustment for confounding. Models utilized wave of data collection as the family variable and study identification number as the link variable. Generalized estimating equations enable analysis of repeated measures over time and take into account the dependent structure of the data, given within-person correlation. The benefits of this approach include accounting for within-subject and within-group correlation and accommodating inconsistent intervals between data points [43]. Factors that were independently associated with food insecurity over time were included in a multivariate longitudinal model that utilized a generalized estimating equation. Stata 13 version 1 was used for analyses (StataCorp LP, College Station, TX, USA) [44].

## 3. Results

Table 1 presents demographic characteristics of the sample (*n* = 683) at each wave. Changes in age, sex, partnership status, and race/ethnicity composition over time were assessed, and findings show there was stability in characteristics over the three time periods (all *p*-values for change >0.05). The sample was 51.5 percent Black, 43 percent White and 2.7 percent Latino. Over 60% of respondents were female. Changes in employment status, income level, and number of moves since Hurricane Katrina were statistically significant over the three waves of data collection. Employment dropped in the fourth wave of follow up, and income and number of moves increased over time.

Food insecurity, disability, mental health, and social support prevalence in the study sample changed significantly over time (Table 2). Food insecurity ranged from 30.4 percent in wave two to 20.1 percent in wave three. In wave four, food insecurity prevalence increased slightly to 23.1 percent. Disability prevalence increased with each subsequent wave of data collection, from 13.4 percent in wave two to 20.5 percent in wave four. Poor mental health and low social support prevalence decreased with each subsequent wave of data collection (47.9 to 38.5 and 24.2 to 15.3 percent, respectively).

Examination of bivariate associations among health, demographic characteristics, and the outcome food insecurity indicated employment, partnership, income, older age (66+), and white race are statistically significant and inversely associated with food insecurity, while female sex, moves since Katrina, disability, poor physical and mental health, and low social support were statistically significant and positively associated with food insecurity (Table 3). 

These factors were included in a generalized estimation equation model for panel data to determine associations with food insecurity two to five years after initial exposure to Hurricane Katrina (Table 4). Respondents who reported having a partner (OR 0.93; 95% CI 0.89, 0.97), higher income ($35–50 K OR 0.89; 0.83, 0.96; >$50 K OR 0.84; 0.77, 0.91), and being White (OR 0.95; 0.91, 1.10) or “other” race (OR 0.84; 0.73, 0.97) were less likely to report food insecurity over a five-year time frame post-disaster. Respondents who were female (OR 1.05; 1.01, 1.10), reported poor physical health (OR 1.08; 1.03, 1.13) or mental health (OR 1.13; 1.09, 1.18), or low social support (OR 1.14; 1.08, 1.20) were more likely to report food insecurity over time.

## 4. Discussion

According to the USDA, average food insecurity prevalence in 2007–2009 in Louisiana and Mississippi was 10.0 percent and 17.1 percent, respectively [45]. We would expect baseline rates of food insecurity in this sample to be similar. We found food insecurity prevalence was 30.4, 21.0, and 23.1 percent in waves two, three, and four of data collection in the present sample, much higher than average state prevalence rates over a similar time frame. However, caution is warranted in comparing food insecurity rates from our study to the National average, since a standardized, validated measure of food insecurity was not included in the G-CAFH study, as the purpose of the study was to more broadly understand child and family health during long-term disaster recovery and it was not focused specifically on food insecurity. However, the high rate of food access issues described in this population make it increasingly important to examine the food environment post-disaster as it is not well understood, and there is little in the literature on the impact of disasters on those families that experience food insecurity during the year or may be living on the edge, and the disruption due to a disaster creates greater household strain.

In this sample of households heavily impacted by Hurricane Katrina, being female, having poor physical and mental health, and low social support were risk factors for food insecurity during long-term disaster recovery, and having a partner, greater income, and being non-Hispanic white or “other” race were protective against food insecurity. This is consistent with food insecurity research that demonstrates that female-headed households, individuals with poor physical or mental health, decline in mothers’ physical or mental health and weaker social ties and emotional support are associated with increased food insecurity [3,4,5,8,9]. It is also consistent with the disaster literature that shows that certain populations are more vulnerable to increased disaster risk and adverse consequences following disasters, such as those with poor physical or mental health, women, and individuals with low social support [21,22,24,28,29,30,34]

The research question and analyses were planned after data collection was completed for waves one through four of the G-CAFH study, therefore the limitations of secondary analysis apply to the present investigation. There was no pre-event data on food insecurity prevalence for this sample due to the unpredictable nature of disasters, therefore we were not able to determine whether food insecurity was a pre-existing issue for families recovering from Hurricane Katrina or a new situation. For this analysis, the inconsistent wording of the food insecurity question may have resulted in different interpretations by the study participants from wave one to waves two and three. Data on this cohort, however, provide a number of distinct benefits that contribute to the existing literature. It was noted earlier that much of the food insecurity research has been conducted with nationally representative samples [17]. This investigation examined a sample of households heavily impacted by a disaster, and starts to tell the story of household food insecurity in a new population. Another strength of this study is in showing a picture of longer-term recovery and food insecurity through a longitudinal study design, which are costly and rare in the disaster literature [46,47]. The study was also carefully designed and executed to enable longitudinal analysis on a cohort, with an 87.6 percent retention rate at wave four of data collection.

To improve disaster outcomes and reduce recovery time, efforts to mitigate, prepare for, and respond to disasters should focus on engagement with vulnerable groups, such as those with physical and mental health distress, female-headed households, and socially isolated populations, to ensure adequate food access and availability. Following disasters, transportation lines may be interrupted, causing access issues for people with physical disabilities and mobility impairments. Further compounding access issues, supply chains may be interrupted, reducing the availability of foods in some areas following disasters [48,49]. Individuals with low social support may lack people to rely on for rides or other help to access foods. For individuals with poor mental health, the additional stress of the disaster experience may exacerbate conditions and result in lower self-efficacy and reduced functioning.

To reduce food insecurity during long-term recovery from disasters, programs and policies should be implemented to increase access to financial support for food or to ensure access to food supplies. One example of such an intervention is the re-issuance of SNAP or WIC benefits to replace spoiled or soiled food supplies and the expansion of benefits to include prepared foods, to enable families living without kitchen facilities to use benefits for meals, as was done following Superstorm Sandy in New Jersey to meet community needs [20]. Systematically adjusting these programs and making the policy known to the end user may reduce uncertainty following disasters and increase utilization. Furthermore, programs that are targeted to reach single-headed, female-headed, low-income, and minority households during non-disaster times with information about securing food in a disaster may reduce vulnerability. Such programs might include educational sessions on food provisions and programs following disasters, facilitation of neighborhood block or community based bulk purchasing of non-perishable foods, or availability of disaster preparedness kits including non-perishable foods through food banks or other food programs. Communicating and building a rapport with community organizations and high risk populations in non-disaster times may also enable more effective post-disaster communication about resources, programs, and services available to affected households. Additional research is needed to determine the effectiveness of policy interventions, such as the re-issued SNAP benefits, for reducing food insecurity post-disaster.

It is also interesting to note that number of moves post-Katrina was not a statistically significant predictor of food insecurity during long-term recovery. The food insecurity literature shows that a change in housing or income stability increases the risk of food insecurity [9]. The present study includes a sample of households heavily impacted by Hurricane Katrina, with families moving an average of 4.59 times at five years post-Katrina. However, in the present analysis, this was not associated with increased food insecurity risk. Additional research on households experiencing food and housing insecurity post-disaster is needed to better understand this circumstance.

This sample was part of a longitudinal study of child and family health following Hurricane Katrina, a group of households heavily impacted or displaced by Hurricane Katrina. This analysis is only a first step towards understanding food insecurity in a post-disaster setting among displaced families, but is not generalizable to all disaster affected populations or the general U.S. population. Additional research is needed on a representative sample of households impacted by disaster and in other geographic locations and hazard types.

## 5. Conclusions

Populations at increased risk for food insecurity also experience increased disaster risk and consequences. Disaster managers and public health practitioners working in these two spheres may be able to find synergy in non-disaster times, as well as when preparing for, responding to, and supporting recovery from disasters. Mitigation of food insecurity in the absence of a disaster may increase resilience to disasters for vulnerable households. Additional research on the experience of households that are food insecure at times during the year and those living with marginal food security, where exposure to a disaster leads them to have low or very low food security, is needed to better understand the health impacts of disaster and how to better meet the needs of this population. A better understanding of the role of housing disruption in a post-disaster setting is also needed to inform policies and programs to mitigate food insecurity for families recovering from disaster.

## Figures and Tables

**Table 1:** Sample Characteristics and significance of change over time.

Sample Characteristics	Wave 2 (2007)	Wave 3 (2008)	Wave 4 (2009–2010)
	*n* (within col %)	*n* (within col %)	*n* (within col %)
**Employed** (20+ h per week) **	335 (45.6)	349 (45.5)	328 (40.0)
**Partnered** (married, living as married)	341 (42.5)	344 (44.5)	372 (44.2)
Income *			
<$10 K	274 (34.3)	224 (29.1)	241 (28.7)
$10–20 K	258 (32.3)	214 (27.8)	265 (31.5)
$20–35 K	126 (15.8)	157 (20.39)	149 (17.7)
$35–50 K	71 (8.9)	88 (11.4)	87 (10.3)
>$50 K	58 (7.3)	68 (8.8)	84 (10.0)
Don’t know/refused	12 (1.5)	19 (2.5)	15 (1.8)
**Age**			
18–34	154 (19.2)	129 (16.7)	142 (16.8)
35–49	272 (34.0)	266 (34.4)	272 (32.2)
50–65	271 (33.8)	271 (35.0)	305 (36.1)
66+	104 (13.0)	108 (14.0)	125 (14.8)
**Number of moves since Katrina** [Mean (SD)] ***	3.79 (2.00)	3.81 (2.04)	4.59 (2.95)
**Race/Ethnicity**		Constant variables	
Black	420 (51.5)		
White	351 (43.0)		
Latino	22 (2.7)		
Other	23 (2.8)		
**Sex**			
Male	305 (39.3)		
Female	471 (60.7)		

* *p* < 0.05, ** *p* < 0.01, *** *p* < 0.001.

**Table 2:** Sample Health Characteristics and significance of change over time ^.

Health Characteristics	Wave 2	Wave 3	Wave 4
	*n* (within col %)	*n* (within col %)	*n* (within col %)
Food insecurity ***	244 (30.4)	163 (21.0)	194 (23.1)
Disabled ***	98 (13.4)	121 (15.8)	173 (20.5)
Poor physical health	405 (50.7)	397 (51.2)	435 (51.7)
Poor mental health ***	383 (47.9)	300 (38.7)	324 (38.5)
Low social support ***	186 (24.2)	129 (18.4)	125 (15.3)

*** *p* < 0.001. ^ *p*-values from chi2 statistic reported for at least one difference between waves.

**Table 3:** Bivariate association between demographic characteristics and health status with food insecurity over time ^.

Demographic and Health Characteristics	Beta Coefficient	Standard Error
**Employed** (20+ h per week) ***	−0.08	0.02
**Partnered** (married, living as married) ***	−0.10	0.02
**Income** (<$10 K)		
$10–20 K **	−0.06	0.02
$20–35 K ***	−0.16	0.03
$35–50 K ***	−0.24	0.03
>$50 K ***	−0.30	0.03
Don’t know/refused	−0.11	0.06
**Age** (18–34)		
35–49	0.02	0.03
50–65	−0.03	0.03
66+ **	−0.11	0.04
**Race/Ethnicity** (Black)		
White **	−0.06	0.02
Latino	0.07	0.07
Other	−0.12	0.07
**Sex** (Male)		
Female ***	0.08	0.02
Moves since Katrina *	0.01	0.004
Disabled ***	0.13	0.02
Poor physical health ***	0.10	0.02
Poor mental health ***	0.17	0.02
Low social support ***	0.17	0.02

* *p* < 0.05, ** *p* < 0.01, *** *p* < 0.001. ^ xtgee models run for each independent variable and the dichotomous outcome food insecurity.

**Table 4:** Odds of reporting food insecurity by demographic and health characteristics of respondents over time.

Demographic and Health Characteristics	Odds Ratio	95% CI
**Employed** (20+ h per week)	1.00	(0.94, 1.04)
**Partnered** (married, living as married) *	0.93	(0.89, 0.97)
**Income** (<$10 K)		
$10–20 K	0.99	(0.94, 1.04)
$20–35 K **	0.92	(0.86, 0.98)
$35–50 K **	0.89	(0.83, 0.96)
>$50 K ***	0.84	(0.77, 0.91)
Don’t know/refused	1.01	(0.85, 1.19)
**Age (18–34)**		
35–49	1.03	(0.97, 1.10)
50–65	0.96	(0.90, 1.02)
66+	0.90	(0.83, 0.98)
**Race/Ethnicity** (Black)		
White *	0.95	(0.91, 0.99)
Latino	1.06	(0.93, 1.21)
Other *	0.84	(0.73, 0.97)
**Sex** (Male)		
Female *	1.05	(1.01, 1.10)
**Moves since Katrina**	1.01	(0.997, 1.01)
**Disabled ^**	1.06	(1.00, 1.13)
Poor physical health **	1.08	(1.03, 1.13)
Poor mental health ***	1.13	(1.09, 1.18)
Low social support ***	1.14	(1.08, 1.20)

* *p* < 0.05, ** *p* < 0.01, *** *p* < 0.001. ^ *p* = 0.05.
